# Retraction: Exosome component 4 promotes epithelial ovarian cancer cell proliferation, migration, and invasion *via* the Wnt pathway

**DOI:** 10.3389/fonc.2026.1885227

**Published:** 2026-06-01

**Authors:** Frontiers Editorial Office

**Keywords:** Epithelial ovarian cancer (EOC), Exosome Component 4 (EXOSC4), invasion, migration, proliferation, Wnt pathway

The journal retracts the article cited above.

Following publication, concerns were raised regarding the image integrity of the published figures. Image overlap concerns were identified in Figure 2A. The authors failed to provide a satisfactory explanation during the investigation, which was conducted in accordance with Frontiers’ policies. As a result, the data and conclusions of the article have been deemed unreliable and the article has been retracted.

This retraction was approved by the Chief Editors of Frontiers in Oncology and the Chief Executive Editor of Frontiers. The authors received a communication regarding the retraction and had a chance to respond. This communication has been recorded by the publisher. Frontiers would like to thank Sholto David for contacting the journal regarding the published article.

